# Optimisation of the preparation phase for orthopaedic surgery: Study protocol for a student-led multimodal prehabilitation feasibility trial (BoneFit)

**DOI:** 10.1371/journal.pone.0314680

**Published:** 2025-02-12

**Authors:** Lee Ingle, Joanna Snook, Lois Smith, Ben Oliver, James Bray, Liz Wells, Jaswinder Moorhouse, Lili Dixon, Phillip Simpson, Selen Osman, John Saxton, Aarthi Rajendran, Ganesh Gopalakrishnan, Tom Symes

**Affiliations:** 1 School of Sport, Exercise & Rehabilitation Science, University of Hull, Hull, United Kingdom; 2 Department of Psychological Services, Hull University Teaching Hospitals NHS Trust, Hull, United Kingdom; 3 Innovation Manager, Hull University Teaching Hospitals NHS Trust, Hull, United Kingdom; 4 Clinical Director for Anaesthetics, Theatres, Critical Care and Pre-Assessment, Hull University Teaching Hospitals NHS Trust, Hull, United Kingdom; 5 Department of Orthopaedics, Clinical Director for Trauma, Hull University Teaching Hospitals NHS Trust, Hull, United Kingdom; IRCCS: IRCCS Ospedale San Raffaele, ITALY

## Abstract

**Background:**

Since the Covid-19 pandemic, a surgical backlog for total hip replacement (THR) and total knee replacement (TKR) surgery remains in the United Kingdom. Multimodal prehabilitation pathways (encompassing exercise, nutritional support and psychological wellbeing) can be utilised to ‘optimise” physical and mental resilience prior to the challenge of surgical intervention. BoneFit is an open-label, non-randomised feasibility trial to determine the recruitment and attendance/adherence rates, delivery and implementation challenges, fidelity, acceptability, and safety of a student-led multimodal prehabilitation intervention in people listed for THR/TKR surgery. We will also determine participant and clinician views of the intervention, and identify any challenges and enablers of inter-institutional partnership working.

**Methods:**

Individuals listed for THR/TKR surgery aged between 18 to 75 years will be assigned to an intervention (n = 25) or usual-care control group (n = 25). The primary outcome measures will be feasibility of delivering the BoneFit intervention. Physical, psychological, quality of life and clinical outcomes will be assessed at three major time-points; T1 (baseline; 2 months from surgery), T2 (2–10 days from surgery), and T3 (3 months following surgery). We aim to show that the trial is feasible and that we can identify a signal of efficacy based on clinical outcomes collected compared to controls. The study was ethically approved by the Health Research Authority (London Bridge Research Ethics Committee: REC reference: 24/PR/0092) in March 2024.

**Discussion:**

The development of a multimodal prehabilitation pathway could improve the physical and mental resilience of individuals awaiting orthopaedic surgery. We aim to determine if this translates to faster discharge and reduced complication rates, thus helping boost surgical throughput and potentially easing surgical backlog. It is likely that the concept of ‘waiting’ lists for surgery should be challenged, rather, individuals should be encouraged to use the time available to ‘prepare’ for surgery.

**Trial registration:**

**Registration details**

ClinicalTrials.gov registration number: NCT06341920.

## Introduction

In the United Kingdom (UK), musculoskeletal conditions account for >25% of surgical procedures undertaken by the National Health Service (NHS) [1]. Total joint arthroplasty / replacement is the most common orthopaedic surgical procedure performed annually, particularly total hip replacement (THR), and total knee replacement (TKR) surgery [2]. The Covid-19 pandemic had a significant deleterious impact on surgical interventions with the NHS pausing elective “non-urgent” surgery in April 2020 [3]. Orthopaedic surgery was viewed as low priority during the pandemic leading to increased waiting times [4], with 7.2 million people listed for elective hospital treatment in January 2023, an increase of 58% since the start of the pandemic [5].

The British Orthopaedic Association [4] calculated that there were 24,000 people waiting in excess of one year for trauma and orthopaedic surgery across the UK, at the height of the pandemic. Orthopaedic surgery is associated with considerable morbidity, increased risk of complications, and excess mortality [1]. From a patient perspective, surgery can lead to a reduction in physical function, a loss of independence due to continued inactivity, immobility and deconditioning. Increased pain and discomfort can lead to physical and mental complications including increased stress, anxiety and depression [6]. These symptoms can lead to higher readmission rates and longer hospital stays [7], especially if individuals are waiting for over one year to receive surgery.

In 2021, the Centre for Perioperative Care published a national position statement for preoperative assessment and optimisation for surgery [8]. Clinical commissioners were urged to establish ‘prehabilitation’ services to support individuals requiring ‘optimisation’ of co-morbidities, nutritional status, psychological preparedness, and physical fitness, thus allowing patients to ‘wait well’ for surgery. Adoption and uptake of these guidelines have been patchy across the UK. Hospital trusts have been encouraged to reconsider the concept of ‘waiting’ lists, and instead consider the period between diagnosis and surgery as ‘preparation’ time [9]. Implementing services allowing individuals to optimise their physical and mental wellbeing prior to surgery will likely lead to improved patient outcomes and could save the NHS money by reducing length of hospital stay, complications and readmission rates [10]. In people requiring orthopaedic surgery, the evidence-base showing the positive impact of prehabilitation on surgical outcomes continues to grow. Recently, a large-scale systematic review and meta-analysis [6] based on 48 unique trials involving 3,570 participants (62% female, mean age 64 years) reported level I moderate-certainty evidence supporting prehabilitation versus usual-care for improving pre-operative function and strength in people undergoing TKR surgery, and moderate-certainty evidence for increased health-related quality of life and muscle strength for individuals undergoing THR surgery [6]. Early intervention is key, as ‘waiting’ list length is likely to be linked to a greater deterioration in function, and a greater challenge for an individual to start making positive lifestyle changes [11]. Low mood and waning motivation can increase whilst ‘waiting’ for surgical intervention which can often exacerbate poor lifestyle choices e.g. increase tobacco use, poorer eating habits, wait gain, and reduced habitual physical activity [12].

Our aim was to introduce the BoneFit trial, an open-label, non-randomised feasibility trial focused on determining the impact of a student-led multimodal prehabilitation intervention on physical and psychological function, quality of life, and clinical outcomes including length of study, complication and readmission rates, in people listed for TKR/THR surgery. We will also determine participant and clinician views of the intervention, and identify any challenges and enablers of inter-institutional partnership working.

## Ethical approval and trial registration

The BoneFit trial was ethically approved by the Health Research Authority (London Bridge Research Ethics Committee: REC reference: 24/PR/0092) in March 2024. The trial sponsor is the University of Hull. The trial was pre-registered at ClinicalTrials.gov (identifier: NCT06341920).

## Methodology

Participants referred to the Department of Orthopaedics at the Hull University Teaching Hospitals NHS Trust (HUTHT) for TKR/THR surgery. BoneFit trial information will be provided to referrals by clinical staff or administrators. Interested parties will contact the Health, Injury and Performance Hub (Hip-Hub) clinic at the University of Hull (https://hiphub.hull.ac.uk/) for an initial appointment.

### Participants

All referrals are awaiting TKR/THR surgery at HUTHT. General inclusion and exclusion criteria are provided below:

### General inclusion criteria

Age 18–75 years;

Waiting for unilateral TKR/THR surgery;

Able to provide informed consent;

### General exclusion criteria

Previous TKR/THR surgery;

Any medical conditions for which moderate to vigorous exercise is contraindicated;

Patellar or hip joint instability;

Any other disease/condition which severely effects functional performance e.g. stroke or Parkinson’s disease;

Chronic depression or significant psychiatric disorder;

Enrolled in another clinical trial (or recently completed one);

Cognitive impairment which would affect compliance;

Patients unable or unwilling to commit to required study follow-ups;

Pregnancy;

### Randomisation and blinding

This is an open-label trial as it would not be possible to mask group allocation (BoneFit intervention versus usual care ‘controls’) from participants or clinical staff administering the interventions. After the initial appointment and following the completion of baseline screening and assessments, participants will be allocated to the intervention or control group by a clinician who is independent to the BoneFit trial. All participants allocated to the BoneFit intervention will initially receive existing early recovery after surgery (ERAS) standard guidance regarding the development of healthier lifestyle choices in preparation for surgery (material delivered by post, or via app and website).

### Data collection and management

Study data will be collected on a case report form by the research team at the point of consent and at each subsequent time-point. Each participant will be allocated a unique study ID number and will remain anonymised for the purposes of the trial. Data will be recorded using both an online system and hard copy data collection sheets and stored securely in the Hip-Hub clinic at the University of Hull.

### Sample size

An *a priori* power calculation to determine sample size was not included as the BoneFit trial has been configured as a feasibility study. However, we did follow statistical guidance indicating that a minimum of 20 participants per group should be included [13]. Therefore, to allow for a potential drop-out rate of approximately 20% (commonly reported in lifestyle interventions), we will attempt to recruit 25 participants to each group (intervention versus control), targeting 50 participants in total.

### Patient and public involvement and engagement (PPIE)

Extensive PPIE work was conducted to inform the separate components of the BoneFit intervention. A patient advisory group including 12 patients aged between 60–80 years (75% male) were interviewed at HUTHT in 2023. We also interviewed clinicians (n = 4; dietician, physiotherapist, occupational therapist, clinical exercise practitioner) based at HUTHT whose roles were to support patients undergoing TKR/THR surgery. Their perspectives and input helped us to develop the selected interventions which have led to the development of BoneFit.

### Study procedures

**Fig 1:**
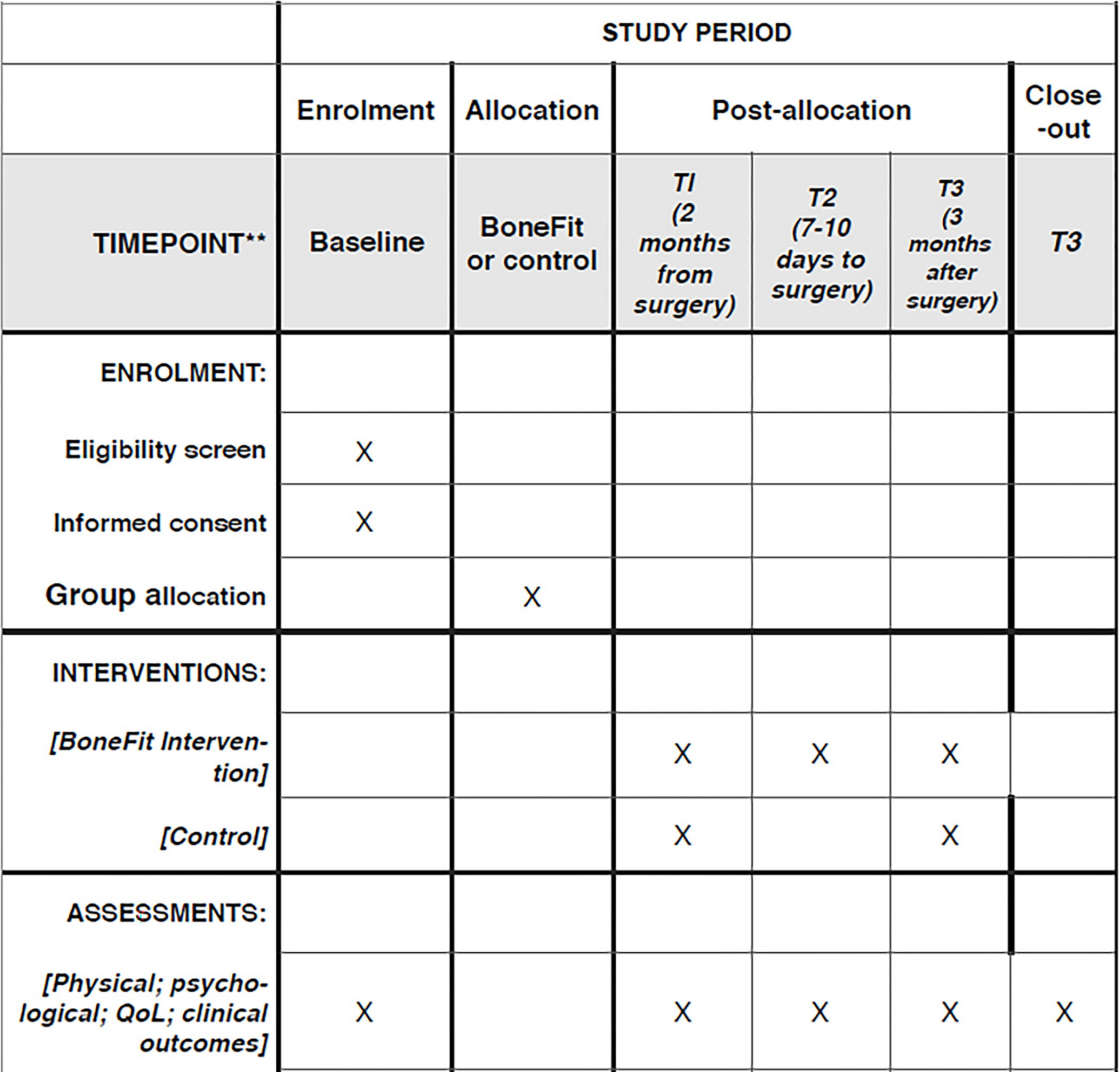
SPIRIT schedule for enrolment, intervention and assessment to the BoneFit feasibility study.

The Hip-Hub clinic offers student-led, patient centred care to local citizens living within the city of Hull. All students are enrolled on undergraduate or postgraduate health-related programmes at the University of Hull. All students work under the guidance of a qualified healthcare professional from their discipline area. To maximise patient engagement and adherence a person-centred approach to behaviour change support is employed [14]. The approach is to focus on enhancing self-efficacy (confidence) in order to engage in new behaviours as well as developing strategies and action plans that meet their priorities and personal circumstances. Approaches will be individualised depending on the level of patient autonomy required to adhere to the PCPs. The schedule for enrolment, intervention and assessment is illustrated in Fig 1.

### Study outcome measures

The primary outcome measures are feasibility and acceptability of the BoneFit intervention. Feasibility will be assessed by determining the number of participants recruited, trained and retained at the end of the intervention, the proportion of sessions delivered and fidelity of delivery. Moreover, participant recruitment, retention and adherence to the intervention will be measured, as well as any adverse events.

Secondary outcomes will attempt to identify a signal of efficacy for changes in physical health (exercise and nutrition), psychological wellbeing, and quality of life compared to usual care. Secondary outcomes for each of the three core components (exercise, nutrition and psychological outcomes) will be evaluated at 3 major time-points (baseline [2 months from surgery], immediately prior to surgery [2 to 10 days], and 3 months following surgery. The Duke Activity Status Index (DASI) [15] will be completed remotely at two extra timepoints (from initial referral) to determine if functional capacity deteriorates before we intervene at the two month point from surgical intervention. We will also evaluate longer-term changes in functional capacity (via DASI) at 12 months following surgery. Clinical outcomes will mainly be assessed at one time-point (3 months following surgery). Controls will be assessed at 2 major time-points for comparative purposes (baseline [2 months from surgery], and 3 months following surgery. Table 1 identifies which outcome measures will be recorded.

**Table 1:** Outcome measures and time-point assessments for individuals recruited to BoneFit.

Variable/Timepoint	From surgical referral	2 months from surgery	2–10 days prior to surgery	3 months post op	12 months post op
**Physical/Exercise**					
DASI	✔	✔	✔	✔	✔
ISWT		✔	✔	✔	
Hand-grip strength		✔	✔	✔	
HOOS-12 or KOOS-12		✔	✔	✔	
**Nutrition**					
MUST		✔	✔	✔	
PG-SGA		✔	✔	✔	
**Clinical Psychology**					
EQ-5D-5L (QoL)		✔	✔	✔	
GAD-7		✔	✔	✔	
PHQ-9		✔	✔	✔	
Emotions thermometer		✔	✔	✔	
**Clinical outcomes**					
Length of hospital stay				✔	
Complications				✔	
Re-admission rates				✔	
VAS pain scale		✔	✔	✔	

DASI—Duke Activity Score Index [15]; ISWT: Incremental shuttle walk test [16]; VAS: Visual analogue scale; MUST: Malnutrition universal screening tool [17]; PG-SGA: Patient-generated global assessment [18]; GAD-7: Generalised anxiety disorder assessment [19]; PHQ-9: Depression test score [20]; Emotions thermometer [21]; EQ-5D-5L: Health and quality of life questionnaire [22]; HOOS-12: Hip disability and osteoarthritis outcome score-12 [23]; KOOS-12: Knee injury and osteoarthritis outcome score-12 [24].

A concurrent mixed methods process evaluation with explore safety, implementation, delivery, and acceptability of the intervention. We will use semi-structured interviews with participants and practitioners along with process data to determine the acceptability of the intervention and to explore barriers and enablers to the implementation of the intervention, interviews will be conducted amongst participants (n = 6), and clinical staff involved in referral and intervention delivery (n = 6). Themes which will be explored will include barriers to recruitment; acceptability and adherence to the intervention (dose received); intervention delivery (fidelity); how the intervention was embedded into clinical practice; safety outcomes: will include adverse events and serious adverse events; assess surgeons’ and surgical practitioners’ willingness to refer to BoneFit; assess participants experiences of the BoneFit intervention.

### Interventions

We were guided by the 2019 NHS Long Term Plan [25], which advocated the development of personalised care plans (PCPs). Validated screening and assessment tools will enable assignment of participants to appropriate levels of support. Those with no increased risk factors and with no increased surgical risk will receive universal support. Further assessment will be undertaken for those requiring more than universal support and they will be allocated to targeted (intermediate risk/needs) or specialist (high risk/complex needs). Individuals may receive different levels of support for the different intervention components: exercise, nutrition and psychological support. Individuals assigned to specialist groups may be excluded from the intervention.

Student-patient interactions will be delivered mainly in a face-to-face individual or group setting. However, in certain circumstances, virtual (one-to-one or group-based using Teams or equivalent) or via telephony may also be offered. Modes of engagement/support will be monitored and recorded as part of the evaluation process. Sessions will mainly be delivered live although some pre-recorded material may be used to supplement live sessions and an online resources library will be developed over time.

### Screening and assessment

Following screening, if an individual (irrespective of allocation to intervention or control group) is identified as being in the specialist group (high risk) or nutrition or psychological support, they would be deemed unsuitable for BoneFit and re-referred for specialist care through usual professional service channels, and their GP would be informed.

#### Physical function

Students will use the Duke Activity Status Index (DASI) [15] to screen for reduced functional capacity. Patients with a DASI score > 34 are at low risk and will be assigned to universal support, those with a DASI score <34 will be referred for an assessment.

Assessment: An incremental shuttle walk test (ISWT) [16] will be performed to assess patients’ functional capacity. Patients with ISWT distance of <475m will be assigned to targeted intervention. Patients with a ISWT distance of <400m or patients with a medical comorbidity that necessitates supervised exercise will be assigned to specialist intervention. If a patient is deemed unsuitable to complete the ISWT by the clinical supervisor due to functional limitations or progressive pain, we will ask them to just undertake a Timed Up and Go test and use this to screen into targeted (<18 seconds), or specialist groups (> = 18 seconds).

#### Nutritional status

We will use the Malnutrition Universal Screening Tool (MUST score) [17] to screen for people at nutritional risk. If an individual scores <1 on MUST, they will be assigned to universal support.

Assessment: Patients scoring >1 but <2 on MUST will be referred to a student nutritionist / dietitian for an assessment which will include using the patient-generated and professional component of the Patient-Generated Subjective Global Assessment (PG-SGA) [18]. They will additionally perform a hand-grip strength test to enable a nutritional diagnosis and direct care in accordance with the Nutrition Care Process mode [26] and will be allocated to the targeted group. If patients score ≥2 on MUST, they will be categorised as ‘specialist’ and be excluded from the BoneFit trial, and be referred to the relevant community dietetics department for further assessment, and their GP informed.

#### Psychological health status

Referrals will be screened by clinic staff using the General Anxiety Disorder Assessment (GAD-7) [19], the Patient Health Questionnaire 9 (PHQ-9) [20], and the ‘need for help’ emotions thermometer [21]. Patients scoring <10 on the GAD-7 or ≤10 PHQ-9 will be assigned to universal support. Patients scoring 15+ on the GAD-7 or 20+ on the PHQ-9 will be categorised as ‘specialist’, and will be excluded from the BoneFit trial, and re-referred to the relevant community mental health services for further assessment, and their GP informed. If immediate risk is identified, ‘high risk’ patients will be referred to their local Crisis Service.

Assessment: Patients will be eligible for the targeted intervention if they score 10–14 on the GAD-7 or 10–19 on the PHQ-9. This will include a psycho-education package with coping exercises and links to video/audio resources. They will also be able to opt-in for 1:1 sessions (maximum of 6 sessions) with trainee clinical psychologist to determine an appropriate intervention based on an individual basis.

Other measures included health-related quality-of-life (HRQoL) assessed by the EuroQoL (EQ-5D-5L) [22], and either the short-form hip disability and osteoarthritis outcome score-12 (HOOS-12) [23], or short-form knee injury and osteoarthritis outcome score-12 (KOOS-12) [24]. Table 2 provides an outline of each intervention. Face-to-face interventions commence at 2 months from surgery, however, participants allocated to the BoneFit intervention arm will receive remote advice (signposting to lifestyle advice/mobile apps of exercise, nutrition and psychological wellbeing) from initial referral.

**Table 2:** Personalised care plans based on group allocation for exercise, nutrition and psychological wellbeing.

	Universal	Targeted	Specialist
**Exercise**	Signposting for home-based exercise programme Increasing frequency, intensity, and duration incrementally to achieve a minimum of 150 min per week of moderate/vigorous physical activity (MVPA) In addition, two sessions of resistance training per week. Sedentary time:replacing daily sitting time with standing/moving time. Smart-phone apps may be considered. Phone call follow ups with student exercise professionals	Group-or individual based training delivered physically or virtually Up to 3 sessions per week (total home and facility-based sessions) of aerobic exercise training (minimum 20 minutes per session)(moderate or high intensity interval training). In addition, two sessions of resistance training per week. Smart-phone apps may be considered.	Delivered face-to-face in student-led clinic Up to 3 sessions per week (including x1 home session) aerobic training (minimum of 20 min per session at moderate intensity. Additionally, two resistance training sessions per week. Close monitoring of signs and symptoms during exercise.
**Nutrition**	Signposting to online/digital dietary advice including basic principles. Pre-recorded offline materials may be available.	Referral to a dietetic/nutrition student for advice on dietary modification, and management. This will be in addition to food fortification advice as per best clinical practice.	Re-referred via GP / specialist community nutritional/dietetics care. Excluded from BoneFit intervention.
**Psychological Support**	Discharge letter and signposting to psychological online/digital support resources (such as NHS talking therapies for anxiety and depression [IAPT]) with opt-in to receive further resources.	Psycho-education package with coping exercises and links to video/audio resources. Option of opt-in for 1:1 sessions (maximum of 6 sessions) with trainee clinical psychologist.	Re-referred via GP / community mental health services. Excluded from BoneFit intervention.

### Statistical analysis

Feasibility outcomes will be reported as percentages and/or counts. Median and inter-quartile ranges will be used to describe the distribution of data. Data distribution assumptions will checked prior to analysis and an intention-to-treat analysis will be conducted with any missing data accounted for using appropriate techniques. Data will be analysed using SPSS (IBM, NY, USA). For quantitative data, normality testing will be conducted and appropriate parametric or non-parametric analysis will be conducted. Qualitative data from semi-structured interviews will be transcribed verbatim and NVivo software (Lumivero, USA) will be used to help explore themes which emanate from the discussions.

### Data monitoring, adverse events and auditing

A trial management steering group made up from key collaborators will meet quarterly to discuss trial progression, data monitoring, trial conduct and safety considerations. Patient representation will also be included in the trial management steering group. Adverse events which may be attributable to the intervention will be monitored by the Hip-Hub manager (JSn) and reported to the management steering group and clinical lead for the trial (TS).

### Dissemination and impact

Throughout the trial, media outlets (including social media) will be informed of progress, and the experiences gained will be presented at national conferences and non-academic outlets such as national governing body publications. On completion, the study results will be published in peer-reviewed journals and presented at scientific meetings.

We hope that the BoneFit intervention will be impactful in a number of areas: 1) informing clinical guidelines and developing the evidence-base around multimodal prehabilitation for individuals requiring orthopaedic surgery; 2) improving local patient care and service delivery through enhancing equity of access to services and building on the principles required to deliver effective, safe services. Supporting people who would benefit from optimisation of co-morbidities and needs-based multimodal PCPs, thereby helping referrals “wait well” and “prepare” for surgery; 3) identifying and delivering education, training and advocacy for student healthcare professionals; 4) local workforce transformation through informing the development of new service pathways and strengthening inter-institutional working relationships. Further funding from national and local/regional sources will be sought if we can identify that the trial shows a signal for improving patient-focused and clinical outcomes. A fully-powered randomised controlled trial protocol would be developed under these circumstances.

In conclusion, whilst waiting lists remain uncomfortably long in some surgical disciplines, as a legacy of the Covid-19 pandemic, healthcare providers can use the ‘waiting’ time as a period of preparation to allow individuals to “optimise’ their physical and psychological function so they are better prepared for the deleterious effects of major surgery. The BoneFit intervention has been designed with the input from patients and clinicians, combined with current NHS guidance. This feasibility study will determine the impact of inter-institutional partnership working, and a student-led clinic designed to improve physical and psychological outcomes, quality of life, and clinical outcomes in people listed for TKR/THR surgery.

## Supporting information

S1 Checklist SPIRIT 2013 checklist: Recommended items to address in a clinical trial protocol and related documents*. (DOCX)

S1 File (PDF)
