# Supplementary material for: Optimisation of the preparation phase for orthopaedic surgery: Study protocol for a student-led multimodal prehabilitation feasibility trial (BoneFit)
Source: PLoS One. 2025 Feb 12;20(2):e0314680. doi: 10.1371/journal.pone.0314680 (PMC11819578; doi:10.1371/journal.pone.0314680)
Supplement: S1 File — (PDF) [file pone.0314680.s002.pdf]

Version number and date: V4, 13-02-2024

IRAS ID: 332551

BoneFit: A Student-Led Multimodal Prehabilitation Service for Orthopaedic Surgical Patients in Hull

Introduction:

In the UK, there are >8 million surgical operations performed every year, and this is likely to continue to rise due to the increasing ageing population [1,2]. Surgery can lead to reduction in physical function, a loss of independence due to continued bed rest and deconditioning, increased pain and discomfort, and can lead to physical and mental complications including increased stress, anxiety and depression. These symptoms can lead to higher readmission rates and longer hospital stays [3].

Due to the recent global pandemic. surgical procedures were cancelled *en masse* leading to increasing waiting lists and, at an individual level, continued deconditioning and an increasing risk of functional limitations particularly in the elderly. In the UK, prehabilitation is a single component of Early Recovery After Surgery (ERAS), a multi-step treatment package designed to help prepare individuals for the physical and mental insult of surgery. Being able to optimise an individual prior to surgery is likely to lead to improved patient outcomes and should save the NHS money by reducing the length of hospital stay, complications and readmission rates [4]. Prehabilitation interventions have evolved over the years becoming multimodal in nature. Interventions typically include exercise, nutrition, and psychological support delivered by a multidisciplinary team [5].

In people receiving orthopaedic surgery, the evidence-base showing the positive impact of prehabilitation continues to grow. In 2023, a large-scale systematic review and meta-analysis based on 48 unique trials involving 3,570 participants (61.5% female, mean age 64 years) reported level I moderate-certainty evidence supporting prehabilitation versus usual-care for improving pre-operative function

and strength in people undergoing total knee replacement (TKR) surgery, and moderate-certainty evidence for increased health-related quality of life (HRQoL) and muscle strength for individuals undergoing total hip replacement (THR) surgery [6].

Aims: The aim of BoneFit is to provide local people living in the Hull region with a needs-based prehabilitation care package (PCP) to improve their physical and mental health, quality of life & reduce their length of hospital stay, complications and readmission rates to hospital following orthopaedic surgery (initially TKR and THR procedures).

Currently, Hull University Teaching Hospitals (HUTH) do not offer a prehabilitation/ ERAS service for local patients. The University of Hull (UoH) will partner with HUTH to provide a student-led multimodal prehabilitation service for referred patients awaiting orthopaedic surgery (initially TKR or THR). This is a proof-of-concept trial where external funding will be applied for to underpin and expand the service to support other specialities, if shown to be successful.

Only low-to-moderate risk patients will be initially referred to receive a PCP (risk score between 0 to 6). Patients who score >=7 points on the operative risk estimation chart will not be referred (see final page of the GP referral form for full details of risk criteria).

Study Start and End-dates

The study is planned to run from March/April 2024 to July 2027. We plan to recruit 50 patients and controls (25 per group). Once these numbers have been achieved we will cease recruitment and just continue with the follow-up visits (final follow up is 12 months following surgery).

- **Inclusion criteria:**

Age 18-75 years;

Waiting for unilateral TKR or THR surgery for a minimum of 6 months;

Able to provide informed consent;

- **Exclusion criteria:**

Previous TKR/THR surgery;

Any medical conditions for which moderate to vigorous exercise is contraindicated;

Patellar or hip joint instability;

Any other disease/condition which severely effects functional performance e.g. stroke or Parkinson's disease;

Age <18 years or >75 years;

No access to smartphone or home internet;

Chronic depression or significant psychiatric disorder;

Enrolled in a clinical trial (or recently completed one);

Cognitive impairment which would affect compliance to BoneFIt service;

Patients unable or unwilling to commit to required study follow-ups and unwilling ness to work with students;

Pregnancy;

- **UoH Student-Led PCPs:**

All students will work under the guidance of a qualified professional based at the UoH. Multimodal prehabilitation involves the core disciplines of exercise, nutrition and clinical psychology. Therefore, students involved in the following programmes will help supply PCPs as part of their student placements, projects/dissertations or as part of an internship:

- BSc Physiotherapy

• BSc Sports Rehabilitation

• MSc Dietetics

• BSc Sports Nutrition

• MSc Clinical Exercise Physiology

• MSc Cardiovascular Rehabilitation

• Clinical Psychology Doctorate

• **Service Design:**

Following guidance from the 2019 NHS Long Term Plan (https://www.-longtermplan.nhs.uk/) we will use a personalised approach when developing the PCPs. Validated screening and assessment tools will enable assignment of participants. Those with no increased risk factors and with no increased surgical risk will receive **universal support**. Further assessment will be undertaken for those requiring more than universal support and they will be allocated to **targeted** (intermediate risk/needs) or **specialist** (high risk/complex needs). Patients may receive different levels of support for the different intervention components: exercise, nutrition and psychological support.

Controls (usual care): In a non-randomised manner, we will identify a group of age- and sex-matched individuals referred for hip (THR) or knee replacement (TKR) surgery from patients who contact the BoneFit service. PIS forms will be circulated by clinical staff to eligible patients awaiting TKR/THR surgery in their routine clinic at Castle Hill Hospital. Interested parties will contact the BoneFit service for more information. If patients agree to participate they will be invited to the University of Hull to provide consent and will undertake a series of baseline assessments and will be allocated by BoneFit staff to the intervention or control group at this point.

Current HUTH waiting list times for orthopaedic surgery is <18 months. Following referral, patients will be asked to complete DASI, download the (enhanced recov-

ery after surgery (ERAS+) app onto their smartphones and follow its guidance. If they do not have access to a smartphone, they will be referred to the ERAS+ website, or if they have no internet connection, hard copies of generic guidance will be posted to them. At the 2-month point prior to surgery, they will be referred to the Hip-Hub for further evaluation and to receive their PCPs.

The Hip-Hub (https://hiphub.hull.ac.uk/)- the UoH sports injuries clinic will manage the patient referral process and BoneFIt will benefit from existing referral, data management, facilities, equipment, and communication systems. Referred patients will initially be contacted by Hip-Hub staff and invited to attend for an initial screening session (see Screening, Assessment and Outcomes sections below). Validated screening tools and outcome measures will be used to determine the type of PCP required, and patient data will be recorded. The type of intervention received will depend on whether a patient requires universal, targeted or specialist PCPs. Students will stay in regular contact with patients to ascertain fidelity and compliance with the PCP.

All students will be working under the support and guidance of trained clinical professionals in physiotherapy, sports therapy, nutrition/dietetics, and clinical psychology or clinical exercise physiology. Student-patient interactions will be delivered either in a face-to-face individual or group setting, virtual (one-to-one or group-based using Teams or equivalent) or via telephone. Sessions will mainly be delivered live although some pre-recorded material will be used to supplement live sessions and an online resources library will be built up over time.

Outcome measures for each of the three core components (exercise, nutrition and clinical psychology) will be evaluated at 5 timepoints (initial surgical referral e.g. 18 months from surgery; baseline [2 months from surgery], immediately prior to surgery [2 to 10 days], and 3 and 12 months after surgery). See Table 1 to identify which outcome measures will be recorded. At initial surgical referral, DASI will be completed remotely. However, the remaining 4 timepoints will require face-to-face interactions and more in-depth investigations.

Table 1. Outcome and timepoints assessments for individuals recruited to BoneFit

Variable/ Timepoint	2 months from surgery	2-10 days prior to surgery	3 months post op	12 months post op
**Physical/ Exercise**				
DASI	✓		✓	✓
ISWT	✓	✓	✓	✓
Timed up and go	✓	✓	✓	✓
Hand-grip strength	✓	✓	✓	✓
HOOS-12 or KOOS-12	✓	✓	✓	✓
**Nutrition**				
MUST	✓	✓	✓	✓
PG-SGA	✓	✓	✓	✓
**Clinical Psychology**				
EQ-5D-5L (QoL)	✓	✓	✓	✓
GAD-7	✓	✓	✓	✓

PHQ-9	✓	✓	✓	✓
Emotions thermomet er	✓	✓	✓	✓
**Clinical outcomes**				
Length of hospital stay			✓	✓
Complicati ons			✓	✓
Re-admission rates			✓	✓
VAS pain scale	✓	✓	✓	✓

DASI - Duke Activity Score Index; ISWT: incremental shuttle walk test; VAS: visual analogue scale; MUST: malnutrition universal screening tool; PG-SGA: patient-generated global assessment; GAD-7: generalised anxiety disorder assessment; PHQ-9: depression test score; EQ-5D-5L: health and quality of life questionnaire; HOOS-12: hip disability and osteoarthritis outcome score-12; KOOS-12: knee injury and osteoarthritis outcome score-12.

At initial surgical referral, all participants referred for BoneFIt will receive existing ERAS guidance on developing healthier lifestyle choices in preparation for surgery

(material delivered by post, or via app and website). It is important to intervene immediately after surgical referral as the longer people sit on a waiting list, the more deconditioned they become, and the harder it is to start making positive lifestyle choices (low activators). Low mood can increase whilst waiting which can often exacerbate poor lifestyle choices e.g. increase tobacco use, poorer eating habits, wait gain, etc.

Study Outcomes:

- **Primary objective**

The primary outcome measures are feasibility and acceptability of the BoneFit intervention. Feasibility will be assessed by determining the number of participants recruited, trained and retained at the end of the intervention, the proportion of intervention sessions delivered and fidelity of delivery. Moreover, participant recruitment, retention and adherence to the intervention will be measured, as well as any adverse events. To determine the acceptability of the intervention and to explore barriers and enablers to the implementation of the intervention, interviews will be conducted amongst participants (n=6), and staff involved in referral and intervention delivery (n=6) (see process evaluation below).

- **Secondary objectives**

- To identify a signal of efficacy for positive changes in physical health (exercise and nutrition), mental wellbeing, and quality of life (using tools described below) compared to usual care;
- To identify differences in DASI, length of hospital stay, complications and readmission rates to hospital following orthopaedic surgery (initially TKR and THR procedures) compared to usual care;
- Assess surgeons' and surgical practitioners' willingness to refer to BoneFIt;
- Assess participants experiences of the BoneFit intervention;

Sample Size and Process Evaluation:

Feasibility studies are typically unpowered, based on similar studies, we will aim to recruit 25 participants to the BoneFit intervention and 25 usual-care controls (n=50).

A concurrent mixed methods process evaluation with explore safety, implementation, delivery, and acceptability of the intervention. We will use semi-structured interviews with participants and practitioners along with process data to assess:

- Barriers to recruitment;
- Acceptability and adherence to the intervention (dose received);
- Intervention delivery (fidelity);
- How the intervention was embedded into clinical practice;
- Safety outcomes: will include adverse events and serious adverse events;
- We will collect quantitative process data on safety, screening and recruitment, fidelity and dose/drop-out which will enable discussions with clinical teams to identify remedial actions to improve recruitment, retention, and fidelity.

We will conduct a pilot feasibility study of the BoneFit intervention which will take approximately 3 years to complete (including all 12-month follow up visits).

Screening and Assessment for Exercise/Fitness/Functional Capacity:

Screening: We will use the Duke Activity Status Index (DASI) [7] to screen for reduced functional capacity. Patients with a DASI score > 34 are at low risk and will be assigned to **universal support,** those with a DASI score <34 will be referred for an assessment.

Assessment: An incremental shuttle walk test (ISWT)[8] will be performed to assess patients' functional capacity. Patients with ISWT distance of <475m will be assigned to **targeted** intervention. Patients with a ISWT distance of <400m or patients with a medical comorbidity that necessitates supervised exercise will be assigned to **specialist** intervention. If a patient is deemed unsuitable to complete the ISWT by the clinical supervisor due to functional limitations or progressive pain, we will ask them to just undertake a Timed Up and Go test and use this to screen into targeted (<18 seconds), or specialist groups (>=18 seconds).

Screening and Assessment for Nutritional Status

Screening: we will use the Malnutrition Universal Screening Tool (MUST score) [9] to identify patients at nutritional risk. If the patient scores <1 on the MUST score, they will be assigned to **universal support.**

Assessment: Patients scoring >1 on MUST will be referred to a student dietitian for a nutrition assessment. This may result in the prescription of oral nutritional supplements. This will include using the patient-generated and professional component of the Patient-Generated Subjective Global Assessment (PG-SGA) [10]. They will additionally perform a hand grip strength test to enable a nutritional diagnosis and direct care in accordance with the Nutrition Care Process mode [11] and will be allocated to **targeted or specialist groups** accordingly.

Screening and Assessment for Psychological Status:

Screening: Patients will be screened using the General Anxiety Disorder Assessment (GAD-7) [12], the Patient Health Questionnaire 9 (PHQ-9) [13] and the 'need for help' emotions thermometer [14]. Patients scoring <9 on the GAD-7 or PHQ-9 and/or <5 on the 'need for help' item of the emotions thermometer will be assigned to **universal support.**

Assessment: Patients scoring >9 on the GAD-7 or PHQ-9, and/or >5 on the 'need for help' item of the emotions thermometer will be invited to speak to a student clinical psychologist to determine **targeted or specialist** allocation.

Based on initial screening, individuals will be categorised into a universal, targeted or specialist group and will receive an intervention based on the criteria identified in Table 2. **Table 2.** Personalised Care Plans Delivered based on Needs Assessment

	Universal	Targeted	Specialist

**Exercise**	Home-based exercise (supported by regular phone/video calls with student physios/ sports rehabilitation therapists);<br><br>Increasing frequency, intensity, and duration incrementally to achieve a minimum of 150 min per week of moderate/ vigorous physical activity (MVPA)<br><br>In addition, 2 sessions of resistance training per week.<br><br>Sedentary time:replacing daily sittingtime with standing/moving time.	Delivered by student physios/ sports rehabilitates group-based training (F2F - attend Hip-Hub sports injuries clinic at UoH or delivered virtually through Teams).<br><br>3 sessions per week (total home and facility-based sessions) of aerobic exercise training (minimum 20 minutes per session) (moderate or HIIT).<br><br>In addition, 2 sessions of resistance training per week.<br><br>Sedentary time:replacing daily sittingtime with standing/moving time (use activity diaries)	Delivered 1:1 in F2F Hip-Hub setting.<br><br>3 sessions per week (including x1 home session) aerobic training (minimum of 20 min per session at moderate intensity.<br><br>Additionally, if able, 2 resistance training sessions per week.

**Nutrition**	Dietary advice including basic principles. Supported by regular phone calls / video calls with student nutritionists.	Dietetic/nutrition counselling/advice with a student dietician, including dietary modification, management of nutrition impact factors and oral nutritional supplements. This will be in addition to food fortification advice as per best clinical practice.	Artificial nutrition support recommended by a dietitian as per best clinical practice. EXCLUDED FROM INTERVENTION.

**Psychological Support**	Signposting to psychological support resources (such as relaxation and mindfulness apps) or as printed resources as appropriate. Supported by regular student-led phone/ video calls	Facilitated peer-group support emphasising mutual problem solving, plus a package of up to 6 1:1 sessions with a student clinical psychologist focusing on self-regulation of stress/anxiety, self compassion and self-soothing skills personalised to individual needs.This is consistent with typical level 2 psychological support practice as per NICE Guidance[64].	Full clinical psychological assessment and individualised intervention plan with a clinical psychologist(or equivalent),as per best clinical practice andl Level 3/4 of NICE guidance [64]. EXCLUDED FROM INTERVENTION

If an individual is identified as being in the Specialist group for Nutrition or Psychological Support they would be re-referred via HUTH for specialist care through usual professional service channels. Nutrition/dietetics/clinical psychology students would not be involved in their future care/treatment.

• **Theoretical/Conceptual Framework:**

To maximise patient engagement and adherence a person-centred approach to behaviour change support will be employed across all components (exercise, nutrition and psychological support) [15]. Students will be encouraged to use a motivational-type interviewing, and adopt the COM-B model of behaviour change [16]. We will focus on enhancing participants' self-efficacy (confidence) to engage in new behaviours as well as developing strategies and action plans that meet their priorities and personal circumstances. Approaches will be individualised depending on the level of patient autonomy required to adhere to the PCPs.

• **Anticipated impact:**

BoneFit will benefit the NHS and patients in 4 ways: 1) informing clinical guidelines and developing the evidence-base around multimodal prehabilitation for patients requiring orthopaedic surgery; 2) improving local patient care and service delivery through enhancing equity of access to services and building on the principles required to deliver effective, safe services. Supporting patients who would benefit from optimisation of co-morbidities and needs-based multimodal PCPs, thereby helping them "wait well" for surgery; 3) identifying and delivering education, training and advocacy for student healthcare professionals; 4) local workforce transformation through informing the development of new service pathways. Further funding from national (NIHR, MRC, orthopaedic charities) and local/regional sources will be sought if we can identify that the pathway is successful in terms of improving patient outcomes.

Identification of participants:

Participants awaiting TKR/THR surgery will be provided with PIS V4 regarding BoneFit during routine clinics at Castle Hill hospital. Contact details of the BoneFIt team are included and interested parties are encouraged to contact the team. Interested parties will be invited to the University of Hull for consenting, baseline assessments, and allocation to the intervention or control groups. Hull University Teaching Hospitals Trust staff are only required to bring the trial to the attention of suitable patients at routine THR and TKR clinics and direct them to the participant information sheets. Interested parties should then contact the BoneFit team.

Arrangements for the collection, use, sharing, security and integrity of participant data:

The BoneFit team at the University of Hull may require limited patient data from NHS organisations including Hull University Teaching Hospitals Trust. All data requested will be anonymised and each patient will receive a unique identification code. The University will only request data that is pertinent to the BoneFit intervention. For example, details of psychological interventions undertaken as a consequence of BoneFit screening into universal, targeted or specialist groups. Information requested for targeted interventions would include number of sessions undertaken, intervention details, and intervention outcomes following initial screening. NHS organisation could provide this information in the form of a brief summary statement and there is certainly no requirement for University staff to receive patient notes. This has been discussed with the Clinical Psychology team at HUTHT.

References:

1 Abbott TEF, Fowler AJ, Dobbs TD, *et al*. Frequency of surgical treatment and related Hospital procedures in the UK: a national ecological study using Hospital episode statistics. *Br J Anaesth* 2017;119:249-57.

2 Etzioni DA, Liu JH, Maggard MA, *et al*. The aging population and its impact on the surgery workforce. *Ann Surg* 2003;238:170–7.

3. Hoogeboom TJ, Dronkers JJ, Hulzebos EHJ, *et al*. Merits of exercise therapy before and after major surgery. *Curr Opin Anaesthesiol* 2014;27:161–6.

4. Ditmyer MM, Topp R, Pifer M. Prehabilitation in preparation for orthopaedic surgery. *Orthopaedic Nursing* 2002;21:43-54.

5. Bolshinsky V, Li MH-G, Ismail H, *et al*. Multimodal prehabilitation programs as a bundle of care in gastrointestinal cancer surgery: a systematic review. *Dis Colon Rectum* 2018;61:124–38.

6. Punnoose A, Claydon-Mueller LS, Weiss O, et al. Prehabilitation for Patients Undergoing Orthopedic Surgery: A Systematic Review and Meta-analysis, J*AMA Network Open* 2023; ;6(4):e238050.

7. Hlatky MA ,Boineau RE ,Higginbotham MB, et al. A brief self-administered questionnaire to determine functional capacity (the DukeActivity Status Index). A*m J Cardiol* 1989;64(10):651-654.

8. Booth S,Adams L.The shuttle walking test:A reproducible method for evaluating the impact of shortness of breath on functional capacity in patients with advanced cancer. *Thorax* 2001;56(2):146-50.

9. The MUST Explanatory Booklet. http://www.bapen.org.uk/pdfs/must/must_explan.pdf [accessed April 2023].

10. PG-SGA web tool. https://pt-global.org/pt-global/ [accessed April 2023].

11. Gillis C, Ha sil L, Kasvis P, et al. Nutrition Care Process Model Approach to Surgical Prehabilitation in Oncology. *Front Nutr* 2021; 24;8:644706.

12. Spitzer RL ,et al A brief measure for assessing generalized anxiety disorder: The GAD-7. Vol.166, *Archives of Internal Medicine* 2006.1092–7.

13. Hinz A,et al. Assessment of depression severity with the PHQ-9 in cancer patients and in the general population.*BMC Psychiatry* http://dx.doi.org/10.1186/s12888-016-0728-6.

14. Mitchell AJ ,Baker-Glenn EA, Park B, et al..Can the Distress Thermometer be improved by additional mood domains? Part I I. What i s the optimal combination of Emotion Thermometers? *Psychooncology*. 2010;19(2):134–40.

15. Grimmett C,Bradbury K, Dalton S. et al.The role of behavioral science for pe rsonalised multimodal prehabilitation in cancer; *Frontiers in Psychology* 2021: 12:

16. Michie S, et al.The Behavior Change Technique Taxonomy (v1) of 93 Hierarchically Clustered Techniques: Building an International Consensus for the Reporting of Behavior Change Interventions. 2021 https://academic.oup.com/abm/article/46/1/81/4563254
